# Preparation and application of macromolecules with a fluorescence effect in polymer processing

**DOI:** 10.1080/15685551.2019.1687082

**Published:** 2019-11-04

**Authors:** Meng-Di Chen, Guo Zhang, Shi-Chang Chen, Xian-Ming Zhang, Wen-Xing Chen

**Affiliations:** National Engineering Laboratory for Textile Fiber Materials and Processing Technology (Zhejiang), Zhejiang Sci-Tech University, Hangzhou, China

**Keywords:** Macromolecule tracer, fluorescence effect, single screw extruder, residence time distribution

## Abstract

In this study, 9-anthracenemethyl methacrylate (AMMA) and styrene (St) as monomers and benzoyl peroxide as an initiator were used to synthesize P(St-co-AMMA), a macromolecule tracer with a fluorescence effect, via free radical copolymerization. A fluorescent online detection device was built on the basis of the principle of fluorescence online detection by using the single-screw extrusion platform of a torque rheometer to explore the effect of the amount of macromolecular tracer and screw speed on the residence time distribution of polystyrene in single-screw extrusion. Fourier transform infrared spectroscopy, ^1^H-NMR, thermal stability, fluorescence properties, and rheological properties show that the resulting product P(St-co-AMMA) has a degree of thermal stability, fluorescence, and rheological properties similar to polystyrene, so this product can be used to characterize the residence time distribution during single-screw extrusion. The amount of macromolecular tracer P(St-co-AMMA) does not affect the residence time distribution of polystyrene during single-screw extrusion processing, meanwhile, the minimum residence time decreases and the residence time distribution becomes narrow as the screw speed increases, that is, the axial mixing capacity of the single-screw extruder decreases as the screw speed increases.

## 1. Introduction

Extrusion processing has a significant role in the field of polymer processing because of its high production efficiency, excellent adaptability, and extensive applications [1–3]. As one of the most basic equipment in high-polymer processing, a single-screw extruder has the characteristics of simple structure, convenient operation, continuous extrusion process, and low maintenance cost, so it has been widely used in the field of polymer molding [4–8]. Residence time distribution refers to the time when material particles remain in a reactor from the starting point to the exit point. During extrusion process, the residence time distribution of the fluid not only characterizes the axial mixing capacity of the extruder but also directly affects the flow time and dispersive capacity of a polymer melt [9–11]; as such, residence time distribution is an important parameter of a polymer in the melt processing of single-screw extruders [12–15]. Residence time can be measured indirectly and directly, usually via a tracer [16,17]. The selected tracer must have two basic conditions, namely, good compatibility and detectability, to represent the residence time distribution of a fluid during the flow. For tracers, detectability is easily achieved, and many types of materials, such as radioactive [18], photosensitive [19], fluorescent [20,21], ultraviolet sensing [22], and electromagnetic materials [23], can be applied to tracers. These materials can also be easily and accurately detected. By contrast, achieving tracer properties similar to fluids is difficult. If a tracer can match the flow state of a fluid under the same conditions (e.g. temperature, pressure, and shear rate), and then residence time distribution is the most accurate. However, in practice, finding highly efficient ready-made tracers is difficult.

Most of tracer-related studies have used small-molecule tracers [24,25]. However, during polymer extrusion, viscosity is greatly affected by temperature and shear rate because of its unique viscoelasticity. Unfortunately, small molecules do not have this property. Thus, small molecules cannot perfectly simulate the flow state of a polymer during extrusion under specific conditions. In other words, small molecules cannot be used as tracers for polymers.

At present, the preparation of macromolecular tracers via chemical synthesis has become a novel method. A benzene ring-containing compound generally has good fluorescence properties and excellent heat resistance if it is successfully grafted in a polymer segment; a macromolecule with fluorescent properties can also be prepared [26,27]. Hu and Kadri [28] used the monomer of a target polymer to copolymerize with 3-isopropenyl-alpha, alpha-dimethylbenzyl isocyanate, inserted a reactive group on the macromolecular chain, and reacted the resulting compound with the monomer 9-(methylamino-methly) anthracene with fluorescent properties. As a result, a macromolecular tracer with fluorescent properties is successfully prepared. Thus, preparing a macromolecular tracer by introducing a group with a specific function to a macromolecular chain is practical.

This study mainly aims to develop a new route to obtain a macromolecular tracer by incorporating anthracene moieties to polymer backbones. Specifically, self-made 9-anthracenemethyl methacrylate (AMMA) and styrene (St) were used as monomers, and benzoyl peroxide (BPO) was utilized as an initiator to obtain a new macromolecular tracer P(St-co-AMMA) with a fluorescence effect via free radical copolymerization [29]. A fluorescence online detection device was built on the single-screw extrusion platform of the torque rheometer on the basis of the principle of online fluorescence detection. The effect of P(St-co-AMMA) and screw speed on the residence time distribution of polystyrene in single-screw extrusion was also investigated.

## 2. Experimental

### 2.1. Materials

St (AR; Yongda Chemical Reagent Company) was used after it was further purified, the polymerization inhibitor and water were removed, and reduced pressure distillation was conducted. 9-Anthracenemethanol (9-AM) (98%) was purchased from J&K Scientific Company. Methacryloyl chloride (95%), chloroform (1.471–1.478 g/mL), and 4-dimethylaminopyridine (DMAP) (95%) were purchased from Sigma-Aldrich. BPO (AR) was purified by dissolving in chloroform, precipitating in methanol, and vacuum drying at room temperature for use. Toluene (AR) was purchased from Hangzhou Gaojing Fine Chemical Company. BPO, pyridine (AR), sodium sulfate, sodium hydroxide, sodium chloride, and deionized water were purchased from Aladdin.

### 2.2. Experimental equipment

All of the experiments were completed at Zhejiang Sci-Tech University. In addition to the conventional instruments used in the laboratory, the following special equipment was used in this test. Rotary evaporator was purchased from Tokyo Physicochemical Company, instrument model N-1100. High-pressure reactors were purchased from Parr Instruction Company, instrument number 4848. Single-screw extruder was purchased from Harbin Harper Electric Technology Company, instrument model RM-200C. Some basic parameters of the single-screw extruder were as follows: screw diameter, 20 mm; length-to-diameter ratio, 25; maximum temperature, 350 °C; maximum speed, 150 min^−1^; and maximum torque, 160 Nm. From the feeding port to the die, the single-screw extruder was divided into four heating zones, and different temperatures could be set in accordance with various experimental schemes for precise temperature control.

### 2.3. Preparation of AMMA

**Figure 1.**
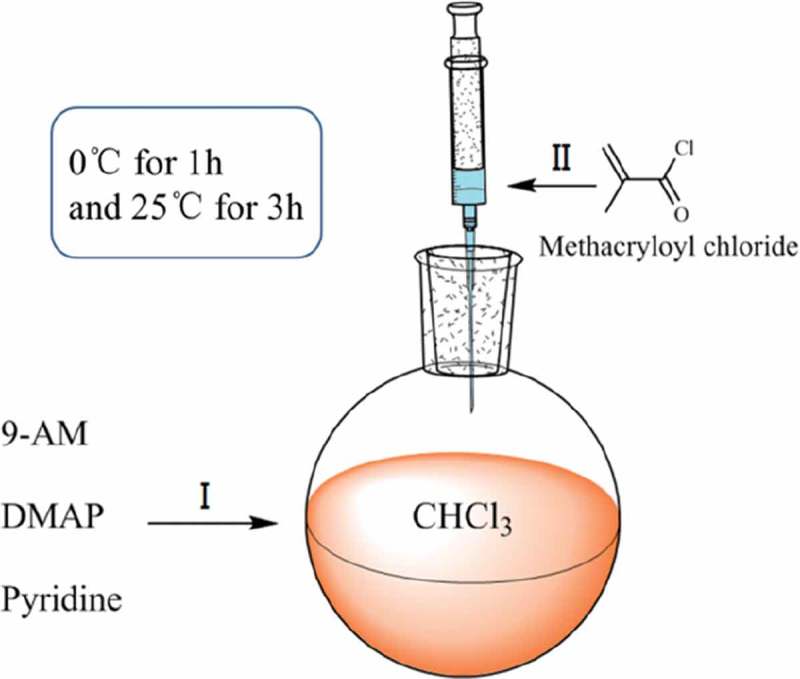
Synthesis route of AMMA.

**Figure 2.**
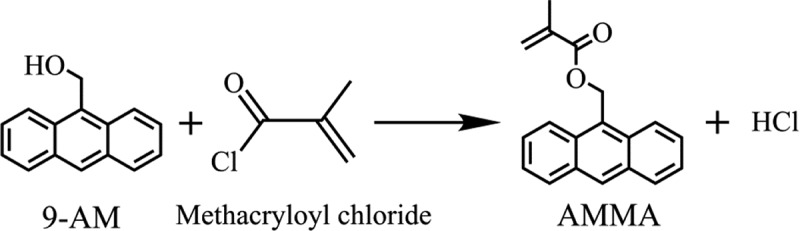
Synthesis of AMMA.

This monomer was prepared via the substitution reaction of methacryloyl chloride and 9-AM. First, 10.0 g of 9-AM and 58.7 mg of DMAP were dispersed in 150 mL toluene. Second, 7.8 mL of pyridine was added to the mixture, and the solution was cooled to 0 °C. Subsequently, 7.5 g of methacryloyl chloride was added dropwise with a syringe to ensure a total addition time of more than 10 min and stirred at 0 °C for 1 h and then at 25 °C for 3 h. Figure 1 shows the schematic of the two aforementioned operational procedures. After the reaction was completed, the reaction product was repeatedly washed at least three times with 1 mol/L HCl, 4 wt% NaOH, and saturated NaCl solution. Finally, the reaction product was dried over Na_2_SO_4_, filtered, and vacuum distilled. The obtained yellow crystallization product was purified through recrystallization from hexamethylene. Figure 2 shows the chemical change that occurs between the monomers during AMMA preparation.

### 2.4. Preparation of P(St-co-AMMA)

**Figure 3.**
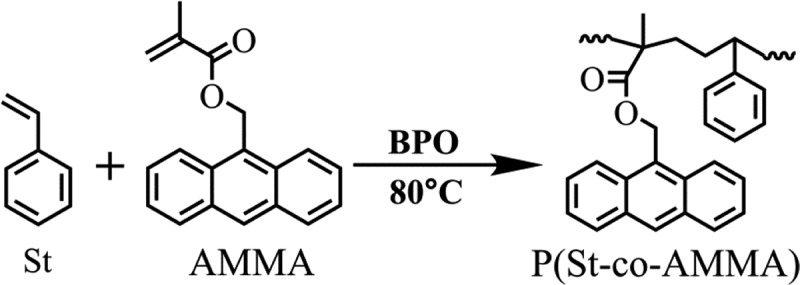
Synthesis of P(St-co-AMMA).

In this experiment, P(St-co-AMMA) was prepared via solution radical polymerization. The composition of the reactants is shown in Table 1. The experiment was conducted in a high-pressure reactor to ensure safety because the reaction time was relatively long. The reaction temperature was 80 °C for 24 h, and nitrogen protection could be accessed. After the reaction was completed, the product was cooled to room temperature, and the reaction solution was slowly poured into an appropriate volume of cold anhydrous methanol (0 °C), which separated the solids from the liquids. The solids were the product. Subsequently, the solid was mixed with an appropriate volume of toluene so that the product could be completely dissolved. This process was repeated at least three times. The solution was ultrasonically washed three times with an appropriate amount of *n*-heptane to ensure the complete removal of the unreacted monomer and initiator. Afterward, the resulting product was dried at room temperature for 5 h, vacuum dried at 30 °C for 12 h, and vacuum dried at 60 °C for 24 h. Figure 3 shows the synthesis of P(St-co-AMMA).

**Table 1.** Composition of reactants for the synthesis of P(St-co-AMMA) copolymer.

	AMMA (g)	Styrene (mL)	Toluene (mL)	BPO (g)	Time (h)
P(St-co-AMMA)-1	2.97	115	170	0.59	24
P(St-co-AMMA)-2	8.91	115	170	0.59	24
P(St-co-AMMA)-3	14.85	115	170	0.59	24

### 2.5. Online detection of residence time distribution in the single screw extruder

#### 2.5.1. Preparation of tracer

First, the polystyrene and P(St-co-AMMA) were mixed at a mass ratio of 100:1 on the mixing platform of the torque rheometer under the following conditions: rotor speed of 45 min^−1^, mixing temperature of 220 °C, and mixing time of 8 min. Second, the mixture was extruded into a strip shape by a 2 mm die of a capillary rheometer and granulated on a granulator to obtain a particle size comparable with polystyrene particles.

#### 2.5.2. Experimental device and measurement process

**Figure 4.**
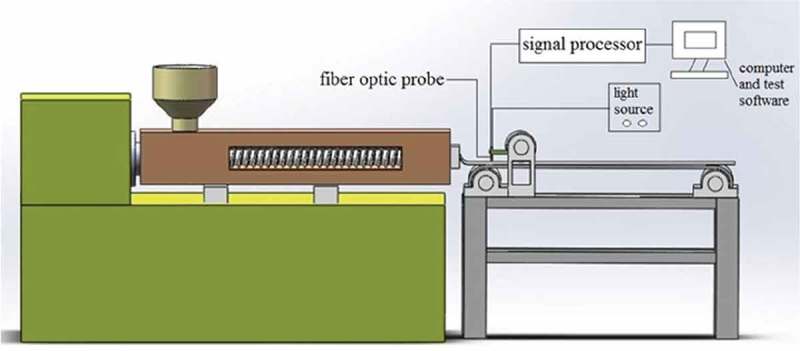
Online detection process of residence time distribution of polystyrene.

Figure 4 shows the online detection process of the residence time distribution of polystyrene in single-screw extrusion. In this experiment, the specific extrusion speed (e.g. 30, 45, and 60 rpm) and the tracer dosage (e.g. 0.01, 0.02, and 0.03 g) were set for a single-screw extruder. The test process began when the baseline of the fluorescence tester was stable and the extrusion process reached a steady state. The amount of polystyrene particles in the hopper was controlled until the screw portion was visible. The tracer was quickly added to the feed port, and the polystyrene particles were added. At the time of adding the tracer (t = 0), the fluorescence online detection device was activated, and the signal was collected at the probe at a frequency of 1 Hz. The baseline intensity should be deducted during signal processing.

## 3. Results and discussion

### 3.1. Verification of the target products

#### 3.1.1. Fourier transform infrared (FT-IR) Analysis

**Figure 5.**
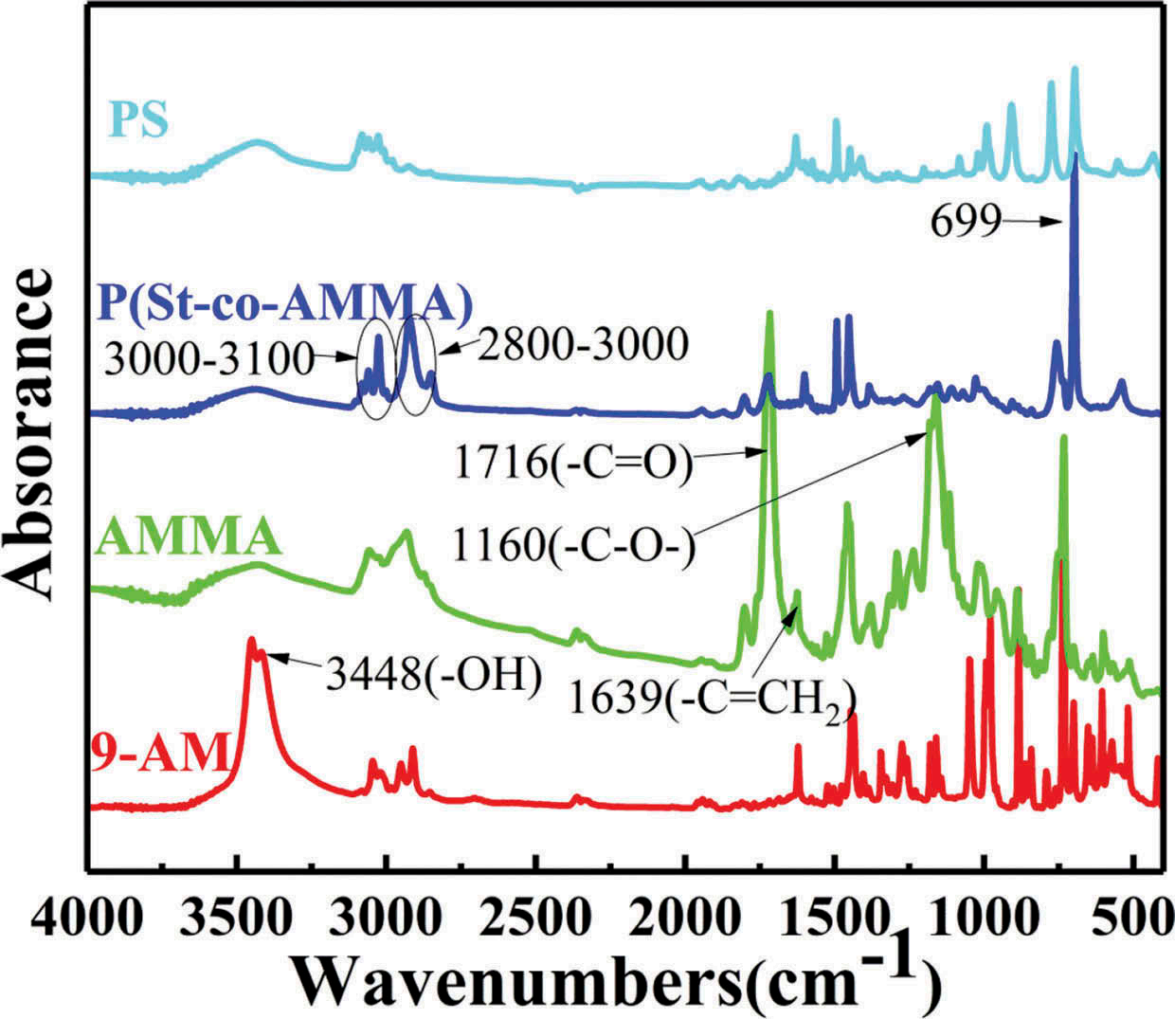
FT-IR spectra of 9-AM, AMMA, and P(St-co-AMMA).

FT-IR spectroscopy was conducted to determine whether the target product was successfully synthesized in this experiment. FT-IR analysis was conducted using a Nicolet 5700 Fourier infrared tester. The measurement range was 4000–400 cm^−1^, the scan rate was 32 s^−1^, and the resolution was 4 cm^−1^. Figure 5 shows an infrared spectrum of 9-AM, AMMA, and P(St-co-AMMA). 9-AM has a strong absorption peak at 3448 cm^−1^, which corresponds to the characteristic stretching vibration peak of the 9-AM hydroxyl group (–OH). The – OH group on 9-AM and the – Cl of methacryloylchloride undergo a substitution reaction; hence, the peak of AMMA at 3448 cm^−1^ disappears, and two new peaks that correspond to the ester group appear at 1716 and 1160 cm^−1^. At the same time, a weak peak, which corresponds to the carbon–carbon double bond in AMMA, is observed at 1639 cm^−1^. The characteristic peak of the two aforementioned functional groups indicates that 9-AM and methacryloylchloride undergo a substitution reaction to form AMMA. In comparison with the infrared spectrum of AMMA, P(St-co-AMMA) has a strong peak at 700 cm^−1^, which corresponds to the monosubstituted group peak of benzene. Moreover, two group peaks, which correspond to the characteristic peaks of saturated hydrocarbon and the stretching vibration peak of C–H in the aromatic ring, appear at 2800–3000 and 3000–3100 cm^−1^, respectively. The appearance of these characteristic group peaks strongly indicates that the intermediate product AMMA reacts with the St monomer to synthesize the target product P(St-co-AMMA). In addition, the ester peak of P(St-co-AMMA) still exists but seems to become less intense, suggesting that AMMA reacts with other monomers and occupies a portion of the product. The most obvious change compared with the infrared spectrum of polystyrene is that P(St-co-AMMA) has a carbonyl peak at 1716 cm^−1^, which also reflects the smooth progress of the reaction from another aspect. Therefore, the obtained final product is the target product P(St-co-AMMA) [30].

#### 3.1.2. ^1^H-NMR Analysis

**Figure 6.**
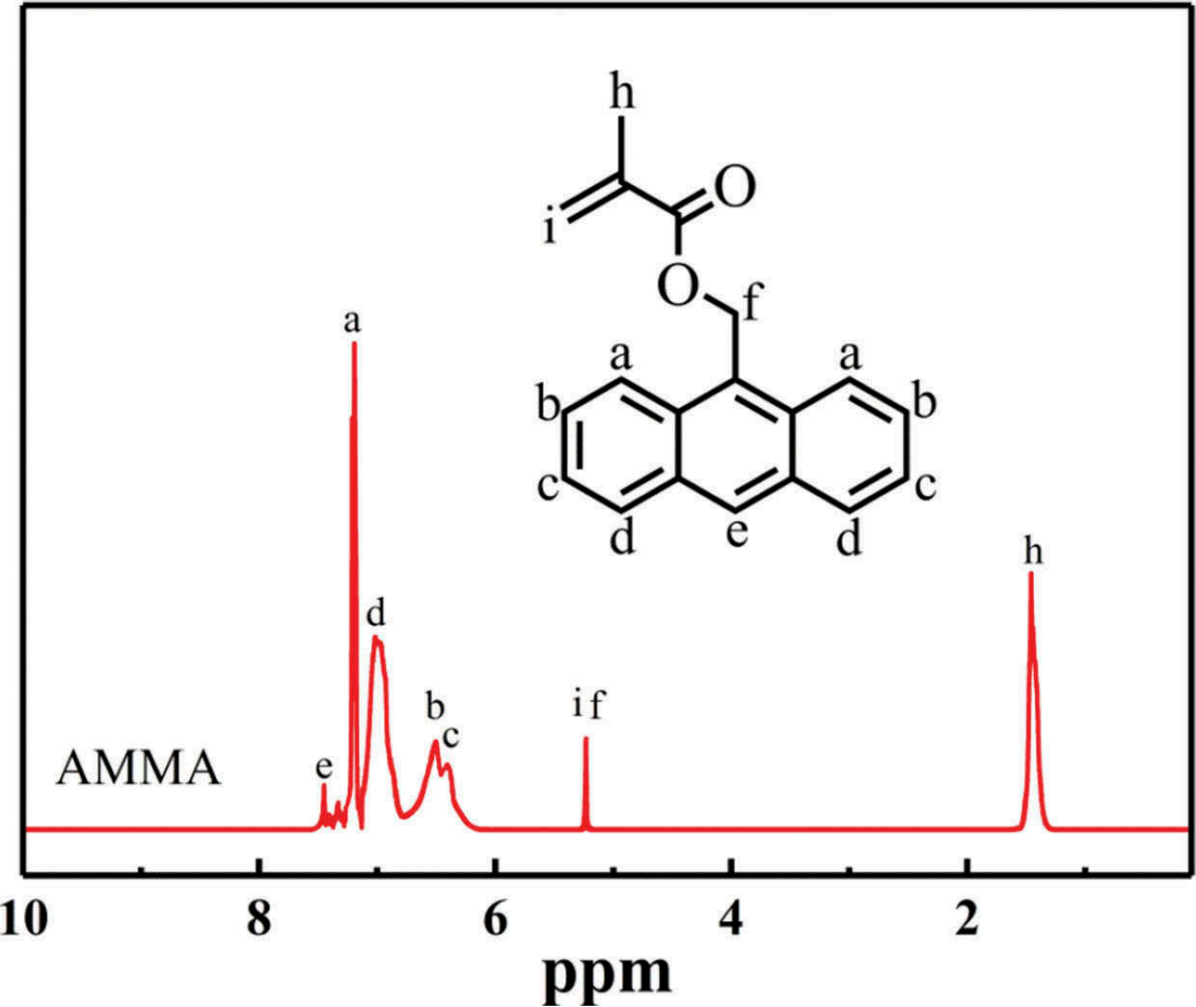
^1^H-NMR spectrum of AMMA.

The chemical structures of AMMA and P(St-co-AMMA) were further characterized by ^1^H-NMR spectrum. In this experiment, the equipment of ^1^H-NMR spectral analysis was Switzerland Bruker Avance DMX400 nuclear magnetic resonance instrument. Deuterated chloroform was used as a solvent for AMMA and P(St-co-AMMA). Tetramethylsilane was the internal standard, and the concentration of the test sample was approximately 2 wt%. Figure 6 shows the ^1^H-NMR spectrum of AMMA. Some peaks attributed to the presence of the anthracene ring are observed at 7.45 (e), 7.21 (a), 7.01 (d), 6.51 (b), and 6.38 ppm (c). A peak is also detected at 5.22 ppm (i) due to the existence of H next to the ester group. Another peak appears at 5.19 ppm (f), which is attributed to the presence of H on the carbon–carbon double bond. Moreover, a peak forms at 1.46 ppm, which is attributed to the existence of H on the methyl group. The synthesized product is AMMA.

**Figure 7.**
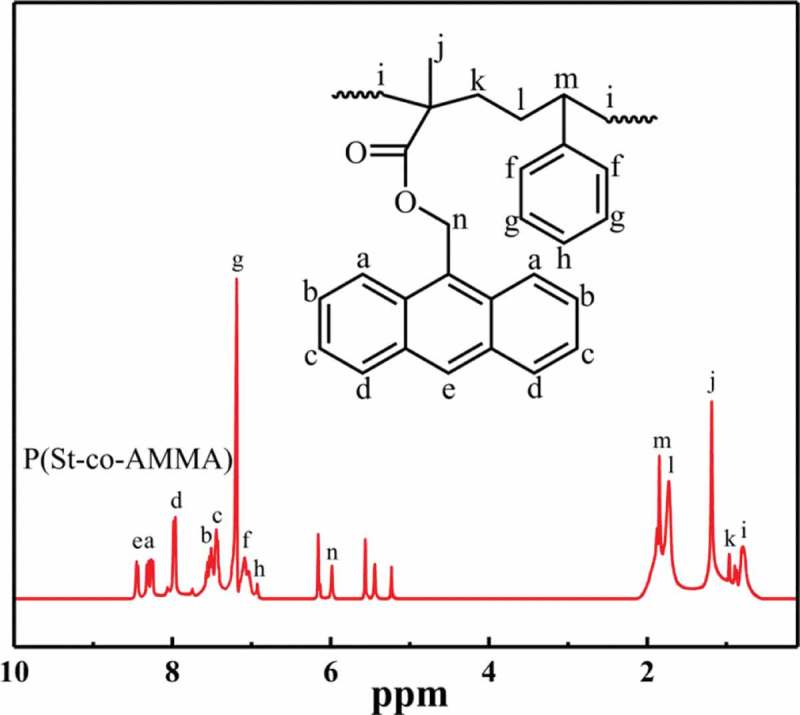
^1^H-NMR spectrum of P(St-co-AMMA).

Figure 7 shows the ^1^H-NMR spectrum of P(St-co-AMMA). Some peaks attributed to the H of anthracene ring appear at 8.45 (e), 8.27 (a), 7.96 (d), 7.52 (b), and 7.45 ppm (c). Some peaks corresponding to the H of benzene ring form at 7.20, 7.10, and 6.93 ppm respectively. A peak is also detected at 5.99(o) due to the existence of H next to the ester group. The peaks at 1.74 (l), 0.98 (k), and 0.78 ppm (i) are due to the presence of methylene. Two peaks are found at 5.45 ppm (p) and 5.24 ppm (n) because of the existence of H on the carbon–carbon double bond. A peak also appears at 1.85 ppm (m) due to the presence of methyne. The ^1^H-NMR spectrum of P(St-co-AMMA) shows that P(St-co-AMMA) is the synthesized product.

### 3.2. Effect of AMMA content on P(St-co-AMMA)

**Figure 8.**
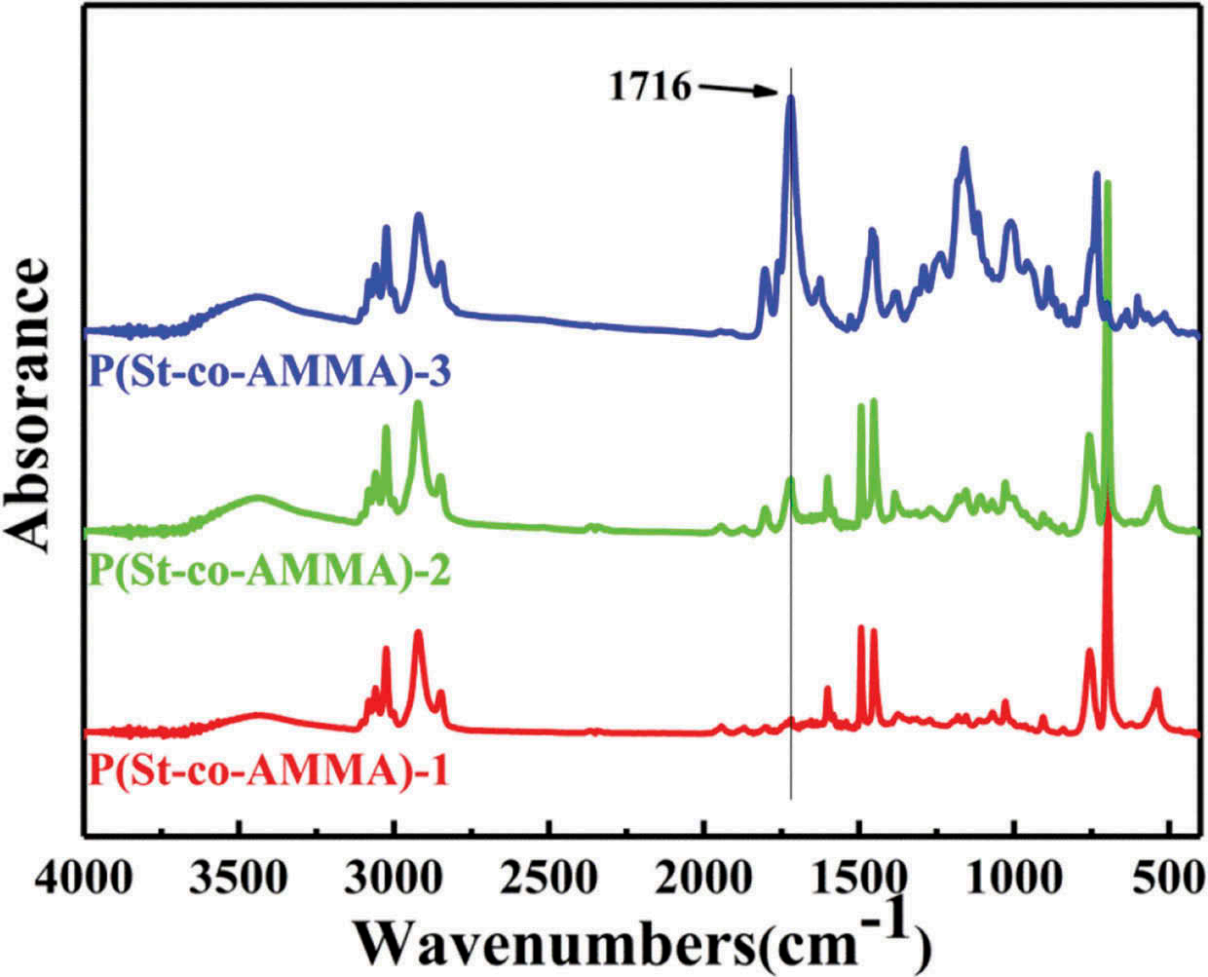
FT-IR spectra of P(St-co-AMMA) with different AMMA contents.

Figure 8 shows a P(St-co-AMMA) infrared spectrum with different AMMA contents. The content of AMMA sequentially increases from P(St-co-AMMA)-1 to P(St-co-AMMA)-3. Figure 8 also reveals that as the AMMA content increases, the intensity of the peak at 1716 cm^−1^ gradually increases, but other characteristic peaks do not change significantly. The amount of St used is consistent in each set of experiments, and the infrared peaks associated with polystyrene are clearly similar. Figure 3 illustrates that the change is caused by the double bond in the two reactants, and the ester group in AMMA remains unchanged during the copolymerization of AMMA with St to form P(St-co-AMMA). Therefore, the amount of AMMA can affect the composition of the final formed product P(St-co-AMMA), thereby changing the content of the ester group in the final product. In summary, this phenomenon is attributed to the increase in the AMMA content, resulting in an increase in the ester group content of the synthesized product.

### 3.3. Thermal stability analysis

**Figure 9.**
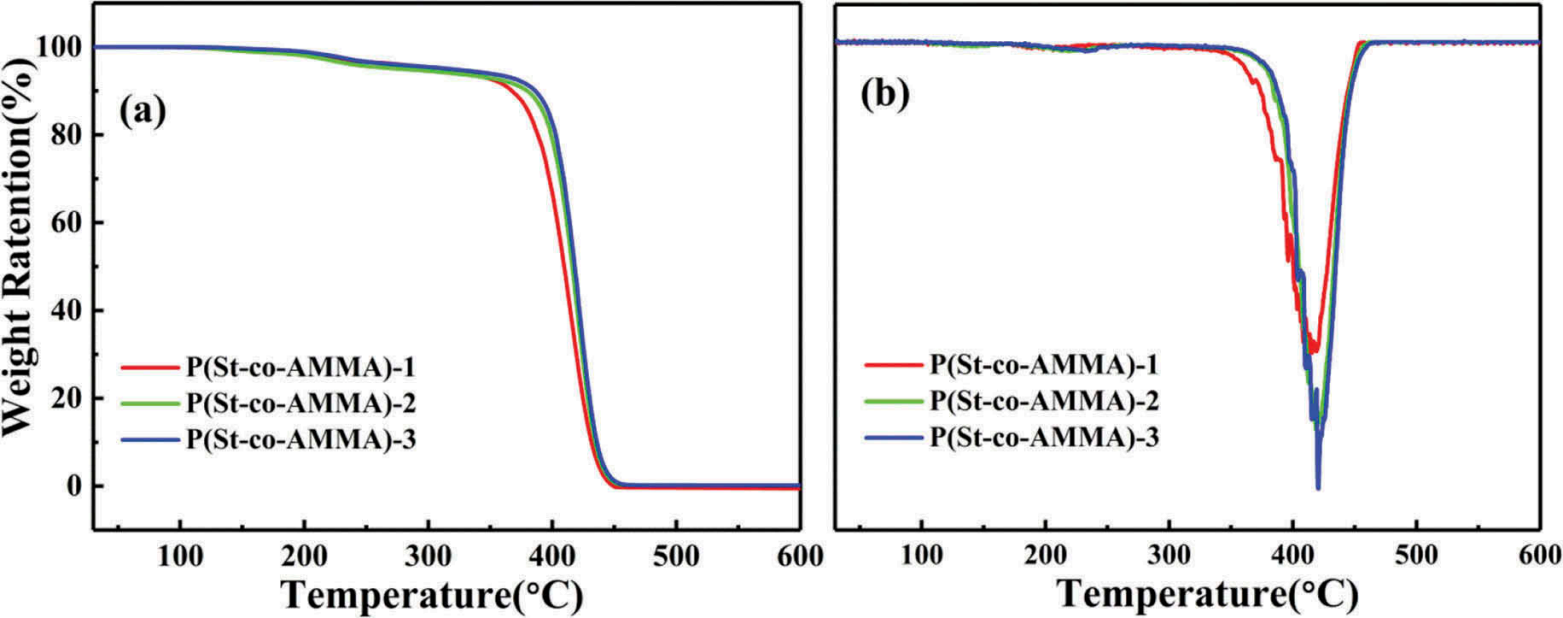
TGA (a) and DTGA (b) curves of P(St-co-AMMA) with different AMMA contents.

The samples were thermogravimetrically analyzed using a Mettler-Toledo thermogravimetric analyzer. The sample to be tested was placed in a drying oven at 100 °C for 12 h to remove moisture, and 5–8 mg of the sample was taken for each test. The test temperature range was 30 °C to 600 °C, the heating rate was 20 °C/min, and the nitrogen flow rate was 40 mL/min. The TGA and DTGA curves of P(St-co-AMMA) with different AMMA contents are shown in Figure 9(a,b), respectively. During the heating process from 150 °C to 360 °C, the quality of P(St-co-AMMA) gradually decreases because of water evaporation. From 360 °C, the quality of P(St-co-AMMA) begins to drop sharply because the main part of the macromolecular chain is still polystyrene, and the degradation temperature of a polystyrene material is approximately 300 °C–400 °C, so this situation is disregarded. In addition, the weight loss rate of P(St-co-AMMA) at 420 °C is the largest, indicating that a large part of the material is degraded at this time (Figure 9(b)). When the temperature reaches 450 °C, the weight loss phenomenon basically disappears, indicating that the tracer P(St-co-AMMA) is completely degraded. The processing temperature of polystyrene is approximately 220 °C, and the decomposition temperature of the tracer synthesized in this subject reaches 360 °C. Consequently, P(St-co-AMMA) can meet its service conditions in single-screw extrusion.

### 3.4. Gel chromatography analysis

Gel chromatography analysis was performed using a gel permeation chromatograph from the Waters Company, USA (model number Waters 1525-2414-2487-717). The GPC test results of the copolymerized product only have one macromolecule elution peak without the elution peak of the small molecules. Thus, the product is only one polymer without byproducts and small molecular residues. Other specific data can be obtained by integrating the test results. The molecular weight and molecular weight distribution of P(St-co-AMMA) with different AMMA contents are presented in Table 2. The molecular weight of P(St-co-AMMA) with various AMMA contents does not greatly differ and slightly increases as the AMMA content increases. Thus, the molecular weight of P(St-co-AMMA) with different AMMA contents does not change greatly. Moreover, the molecular weight distribution index of P(St-co-AMMA) gradually increases as the content of AMMA increases because the input amount of St in the reactants is constant. The addition of AMMA makes the molecular weight of the macromolecular tracer more heterogeneous.

**Table 2.** Molecular weight and molecular weight distribution of P(St-co-AMMA) with different AMMA contents.

	P(St-co-AMMA)-1	P(St-co-AMMA)-2	P(St-co-AMMA)-3
Mn	3.6 × 10^4^	3.0 × 10^4^	3.8 × 10^4^
Mw	7.4 × 10^4^	6.8 × 10^4^	8.8 × 10^4^
D(Mw/Mn)	2.05	2.21	2.31

### 3.5. Fluorescence properties

**Figure 10.**
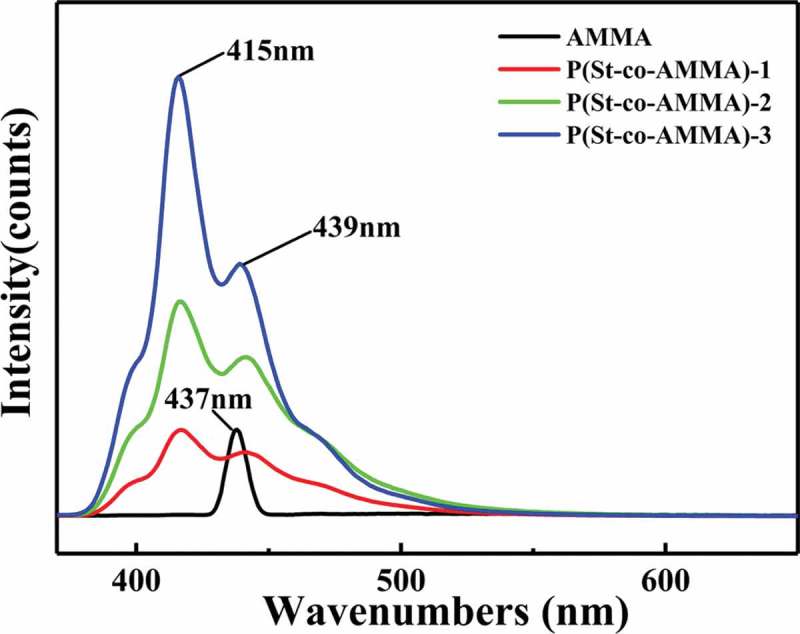
Fluorescence spectrum of AMMA and P(St-co-AMMA) with different AMMA contents.

The samples in this experiment were tested in terms of their fluorescence properties by using an F-46,001 fluorescence spectrophotometer (Hitachi, Japan). During this test, the incident wavelength was 360 nm, the scanning speed was 240 nm/min, the excitation grating was 10.0 nm, and the radiation grating was 5.0 nm. Substances that contain fluorophores likely emit fluorescence, whereas substances that fluoresce generally contain a conjugation structure in which benzene ring and biphenyl structures are the most common. Figure 10 shows the fluorescence spectrum of AMMA and P(St-co-AMMA) with different AMMA contents. A strong peak of AMMA appears at 437 nm. In AMMA, only the anthracene ring fluoresces after ultraviolet light is absorbed. Therefore, in the fluorescence curve of AMMA, the only peak is produced by the anthracene ring. Two excitation peaks also form at 439 and 415 nm on P(St-co-AMMA), the fluorescence peak of the anthracene ring shifts is due to the copolymerization with St. Therefore, the fluorescence peak at 439 nm is caused by the benzene ring in the macromolecular tracer P(St-co-AMMA), and the peak at 415 nm is the fluorescent peak of the anthracene in the macromolecular chain [31]. In the three groups of the fluorescence curves of P(St-co-AMMA), the fluorescence intensity of the macromolecular tracer increases from P(St-co-AMMA)-1 to P(St-co-AMMA)-3 mainly because the reaction amount of AMMA gradually increases from P(St-co-AMMA)-1 to P(St-co-AMMA)-3, whereas the reaction amount of St is always consistent. As a result, the composition of AMMA in the final product P(St-co-AMMA) increases, and the fluorescence intensity gradually increases.

### 3.6. Rheological properties

**Figure 11.**
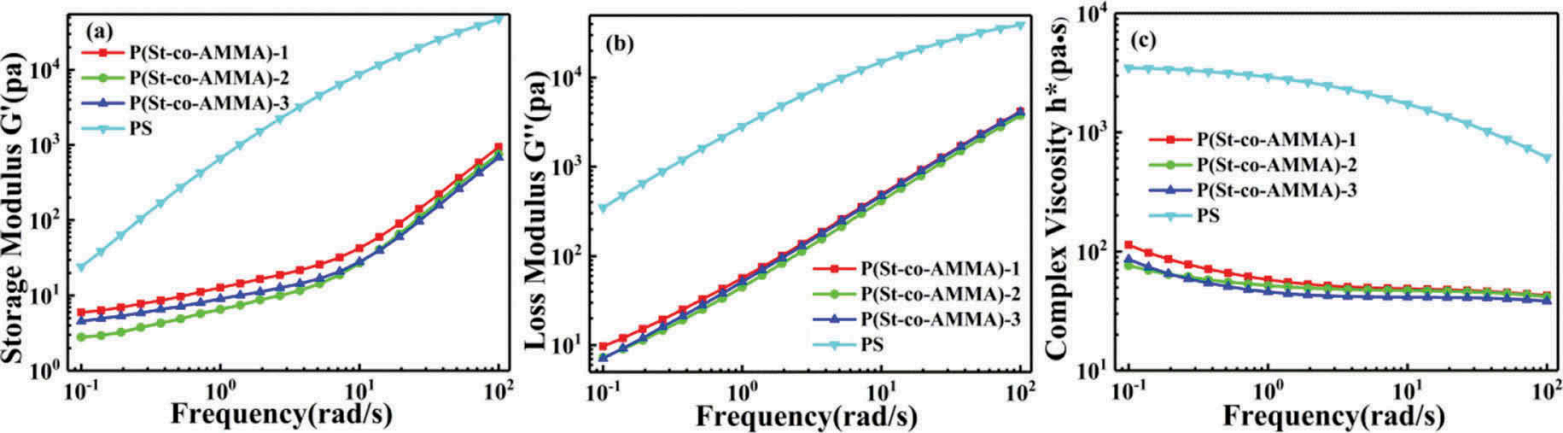
Linear viscoelastic data of P(St-co-AMMA) with different AMMA contents and polystyrene. (a) Storage modulus, (b) loss modulus, and (c) complex viscosity.

The rheological properties of P(St-co-AMMA) were tested on an Anton Paar MCR 301 rheometer. The sample was pressed into a circular sheet with a diameter of 25 mm and a thickness of 2 mm by using the press vulcanizer to enable the direct testing on a rheometer. The pressing temperature was 180 °C, the test temperature was 200 °C, and the shear rate was 0.1 s^−1^ to 100 s^−1^. Figure 11(a), 11(b), and 11(c) illustrate the graphs of the storage modulus(a), loss modulus(b), and complex viscosity(c) of the P(St-co-AMMA) with different AMMA contents and polystyrene as a function of shear frequency. The storage modulus and the loss modulus increase as shear frequency increases over the entire test range, whereas the complex viscosity decreases as shear frequency increases. These trends are the same as those in polystyrene. The difference is that the data of polystyrene are larger than those of P(St-co-AMMA). For the change in the shear rate, the response of polystyrene seems to be more obvious than that of P(St-co-AMMA) because of the relatively large molecular weight of polystyrene. In comparison with small molecules, P(St-co-AMMA) and polystyrene have a similar non-Newtonian pseudoplastic fluid flow state. As a result, P(St-co-AMMA) can accurately characterize the residence time distribution of the single-screw extruder.

**Figure 12.**
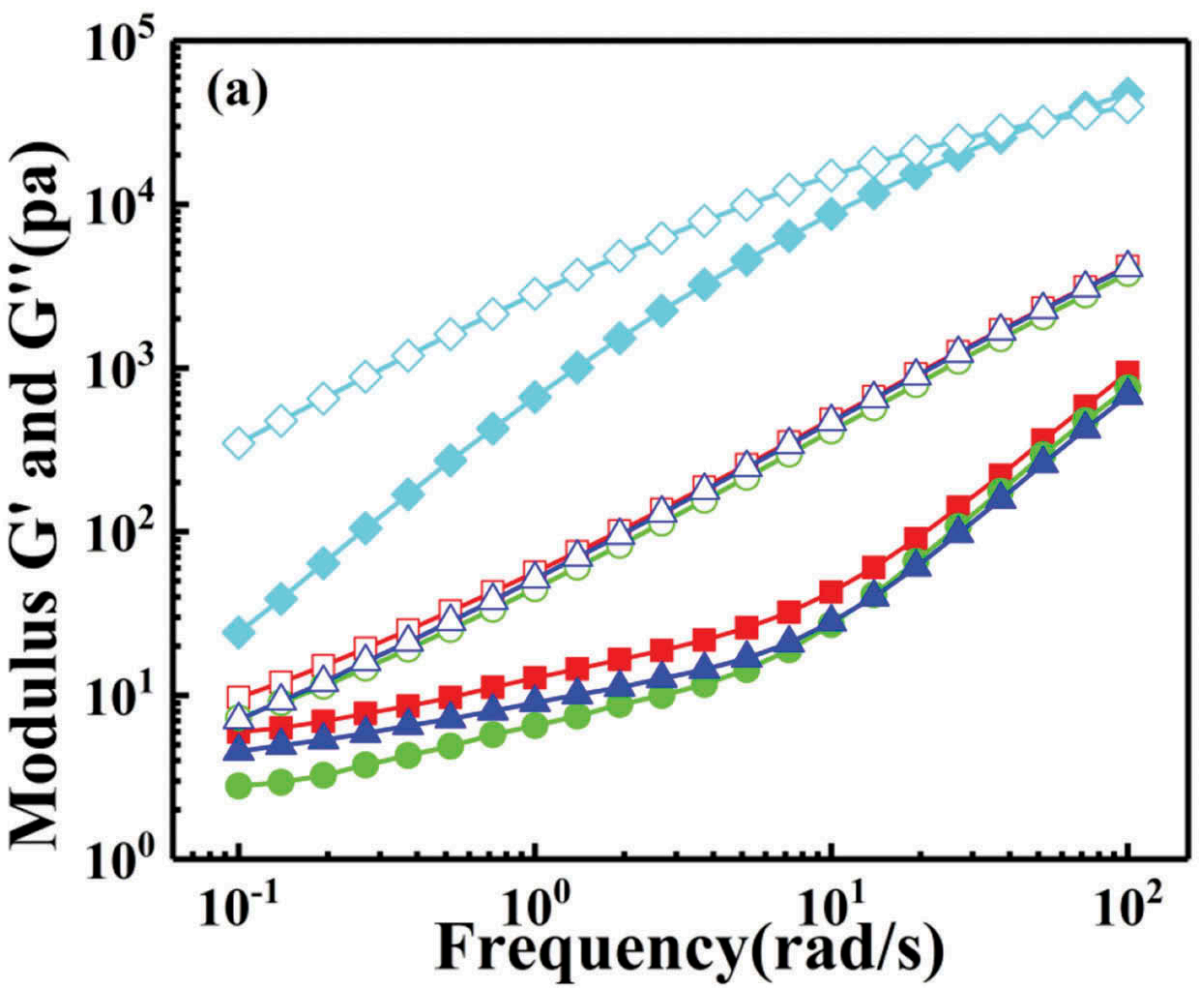
The measurements of the storage and loss moduli (G’ and G”) as a function of applied angular frequency for polystyrene and P(St-co-AMMA): polystyrene (black square), P(St-co-AMMA)-1 (black circle), P(St-co-AMMA)-2 (black up-pointing triangle), P(St-co-AMMA)-3 (Black diamond). Filled symbols correspond to G’ while hollow symbols correspond to G’’.

The storage modulus and loss modulus of polystyrene and macromolecular tracers P(St-co-AMMA) were put together as shown in Figure 12, for all we know the cross-over point, which is the point of intersection of the G’ and G’’, can be used to estimate the relaxation time of the fluid [32]. As is shown in the graph the relaxation time of polystyrene can be clearly seen, in contrast, there is no relaxation time of P(St-co-AMMA) in the frequency range of this experiment. However, according to the trend of the lines, it can be seen that the relaxation time of the macromolecular tracer occurs in the high frequency region. According to the data of the intersection point in the figure, we can obtain that at 200 °C, the relaxation time of polystyrene is λ = 0.02s, and the relaxation time of the macromolecular tracer is estimated to be λ = 0.003s, the difference between two relaxation times is within an order of magnitude. Consequently, the rheological properties of polystyrene and the prepared macromolecular tracer are very similar during high temperature extrusion process, and this conclusion is also consistent with previous experimental results.

### 3.7. Application of polymer tracer in the extrusion process

The concept of the probability distribution can be used to quantitatively describe the residence time distribution of a material in a reactor because the process of extruding a material from a single-screw extruder is continuous and random. Two kinds of probability distribution functions, namely, the residence time distribution density function and the cumulative residence time distribution function, describe the residence time [33]. The residence time distribution density function, also known as the E function, is usually denoted by E(t):

(1) $$E(t)=\frac{c(t)}{\int_{0}^{\infty }cdt}=\frac{c(t)}{\sum_{i}^{\infty }c(t)\Delta t}$$

The cumulative residence time distribution function, also known as the F function, is usually represented by F(t):

(2) $$F(t)=\frac{\int_{0}^{t}c(\tau )d\tau }{\int_{0}^{\infty }c(\tau )d\tau },$$

where c(t) is the voltage signal strength of the tracer at the single screw extruder, and t = 0 when the tracer is added from the feed port.

#### 3.7.1. Effect of the amount of tracer

**Figure 13.**
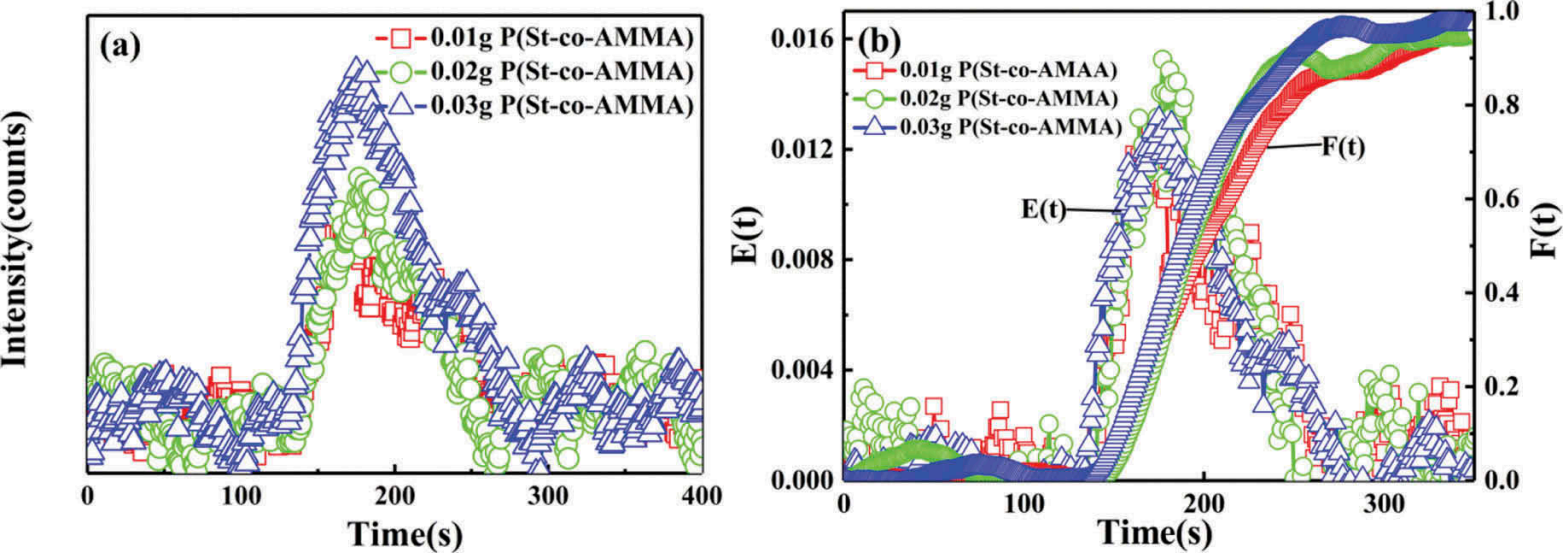
Voltage signal intensity spectrum of P(St-co-AMMA) (a) and residence time distribution spectrum of polystyrene (b) with different tracer contents.

Figure 13(a) presents a graph of the changes in the voltage signal intensity of different P(St-co-AMMA) contents with time in single-screw extrusion, and Figure 13(b) illustrates the residence time distribution density function and the cumulative residence time distribution function obtained after processing in Figure 13(a). In Figure 13(a), the voltage signal intensity of P(St-co-AMMA) increases as the P(St-co-AMMA) content increases. In Figure 13(b), the residence time distribution curves obtained by polystyrene with different tracer contents during the single-screw extrusion process overlap well, indicating that the tracer content has no effect on the residence time distribution of the polystyrene. Furthermore, the amount of tracer has no effect on the axial mixing ability of the single-screw extruder.

#### 3.7.2. Effect of screw speed

**Figure 14.**
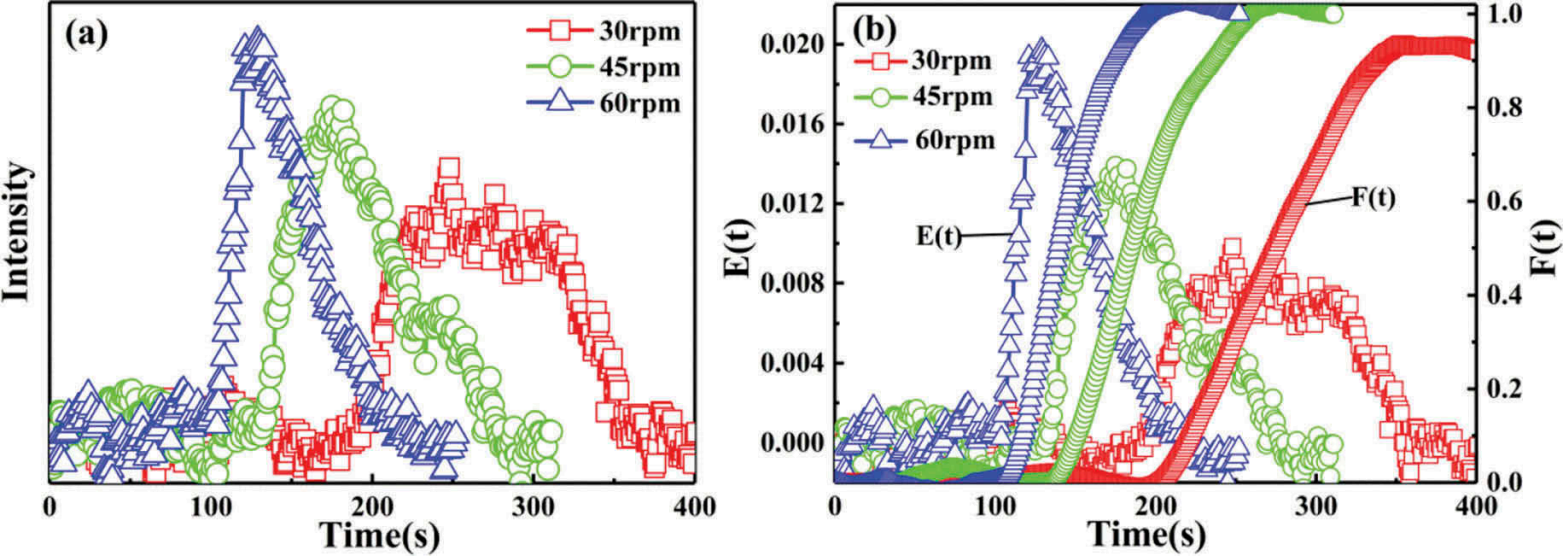
Voltage signal intensity spectrum of P(St-co-AMMA) (a) and residence time distribution spectrum of polystyrene (b) with different screw speeds.

Figure 14(a) shows the voltage signal intensity of the tracer as a function of time under different screw speed conditions, and Figure 14(b) illustrates the plot of the residence time distribution obtained after treatment at different screw speed conditions. In Figure 14(a), the screw speed significantly affects the voltage signal strength, that is, fast screw speeds correspond to a strong voltage signal. Fast screw speed also corresponds to high amount of tracer extruded per unit time. The voltage signal strength also increases. In Figure 13(a), the voltage signal of 0.03 g of P(St-co-AMMA) is strong and clear under the same screw rotation speed and test temperature conditions. Therefore, in terms of the effect of screw speed on the residence time distribution of polystyrene, the determined amount of tracer P(St-co-AMMA) was 0.03 g. In Figure 14, the screw speed significantly affects the residence time distribution of polystyrene in a single-screw extruder; that is, the faster the screw speed is, the narrower the residence time distribution of polystyrene will be, indicating that the axial mixing capacity of the single-screw extruder decreases as the screw speed increases [34].

## 4. Conclusions

In this experiment, a fluorescent macromolecular tracer P(St-co-AMMA) was successfully synthesized via free radical polymerization. The same mechanism as in most macromolecules was developed on the basis of the principle of polymerization by introducing specific groups into the macromolecular chain to obtain a functionalized macromolecular chain. However, this experiment had some differences. A double bond, which can be polymerized, was introduced to a small functional molecule and then copolymerized with a St monomer to prepare a random copolymer. The target product P(St-co-AMMA) was detected and analyzed in terms of three aspects:

1. The structure of each step product was analyzed through FT-IR spectroscopy and nuclear magnetic resonance spectroscopy, and the accuracy of the synthesized product was determined.
2. Fluorescence property detection indicated that the synthesized product P(St-co-AMMA) could emit fluorescence under specific conditions, indicating that P(St-co-AMMA) had the most basic conditions as a tracer.
3. Thermal stability analysis indicated that the synthesized product P(St-co-AMMA) had certain thermal stability. Rheological property analysis showed that the synthesized product P(St-co-AMMA) had rheological properties similar to those of polystyrene. The results of these two experiments demonstrated that P(St-co-AMMA) could act as a tracer to indicate the residence time distribution of polystyrene in a single-screw extruder.

Subsequently, the macromolecular tracer P(St-co-AMMA) was used to characterize the residence time distribution of polystyrene in a single-screw extruder. In this test, the effect of the amount of tracer added and the screw speed on the residence time distribution was mainly discussed. Through a series of experiments, the added amount of the tracer did not affect the residence time distribution of polystyrene in the single-screw extruder, the difference in screw speed changed the residence time distribution, and the screw speed influenced the mixing ability of the single-screw extruder.
